# Supplementary material for: Exploring the Impact of Backward and Forward Locomotor Treadmill Training in Chronic Stroke Survivors with Severe Post-Stroke Walking Impairment: A Single-Center Pilot Randomized Controlled Trial
Source: Brain Sci. 2025 Apr 24;15(5):437. doi: 10.3390/brainsci15050437 (PMC12110432; doi:10.3390/brainsci15050437)
Supplement: Supplementary file 1 [file brainsci-15-00437-s001.zip › brainsci-3560756-supplementary.pdf]

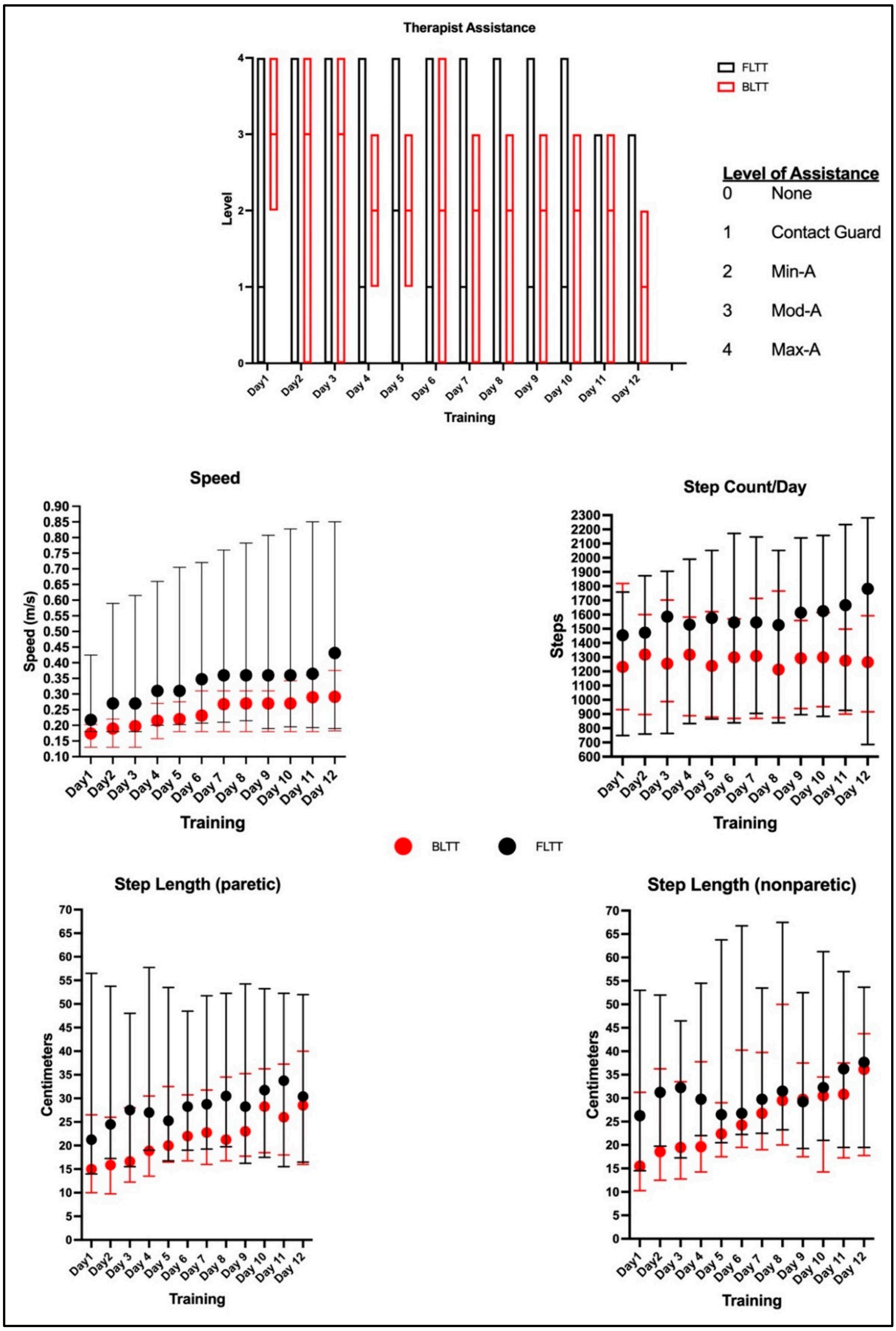

Supplementary Figure S1. Change in training assistance level, training speed, step count, and paretic and non-paretic step lengths over 12 training sessions. BLTT (backward locomotor treadmill training); FLTT (forward locomotor treadmill training). Values are presented as median and range (min-max).

Supplementary Table S1. Safety and Tolerability Questionnaire (Scale 1-10).

BLTT	Headache	Neck Pain	Soreness	Fatigue	Falls	Other/Comments
1*	0	0	0	5	N/A	
2*	0	0	1	6	N/A	
3	0	1	1	0	N/A	
4	0	0	0	0	N/A	
5	0	0	7†	3	N/A	†Soreness was in in back
6	0	0	0	0	N/A	
7	0	0	4	4	N/A	
**FLTT**	**Headache**	**Neck Pain**	**Soreness**	**Fatigue**		**Other/Comments**
1	0	0	7†	3	N/A	†Soreness was in arm from holding the treadmill railing
2	0	0	3	0	N/A	
3	0	0	2	3	N/A	
4	0	0	0	1	N/A	
5	1	4	2	2	N/A	
6*	0	0	1	8	N/A	
7	0	0	0	3	N/A	

The questionnaire was administered following 12 sessions of training at 24hr POST. 0 (None)-10 (Severe). Backward locomotor treadmill training); FLTT (forward locomotor treadmill training). *Subjects BLTT 1 and 2 were poor responders on the 10-meter walk test. Subjects BLTT 1 and FLTT 6 were poor responders on the 3-meter timed up-and-go.

Supplementary Table S2. Individual Level Changes in Spatiotemporal Symmetry.

	Step Length Symmetry (Spatial)				% Single Support Time Symmetry (Temporal)			
**BLTT**	**PRE**	**24hr POST**	**Day 30 POST**	**Day 90 POST**	**PRE**	**24hr POST**	**Day 30 POST**	**Day 90 POST**
**1***	88.1	91.9	96.7	89.2	65.3	61.6	63.0	67.1
**2***	84.1	92.4	95.5	96.8	89.3	94.8	94.5	89.9
**3**	74.6	90.5	78.4	83.1	73.6	77.4	71.5	77.6
**4**	85.4	82.8	84.8	84.5	84.7	94.4	87.7	84.5
**5**	71.9	64.0	57.3	60.7	68.1	68.2	69.3	66.5
**6**	69.4	66.1	74.5	N/A	84.4	86.6	77.8	N/A
**7**	86.2	89.4	89.9	92.6	83.9	86.7	81.8	90.1
**FLTT**	**PRE**	**24hr POST**	**Day 30 POST**	**Day 90 POST**	**PRE**	**24hr POST**	**Day 30 POST**	**Day 90 POST**
**1**	97.2	94.5	95.7	96.4	98.9	88.3	80.5	95.2
**2**	89.3	90.9	87.5	88.1	70.1	74.0	71.5	74.8
**3**	46.1	49.5	49.2	56.4	74.6	72.5	72.9	72.8
**4**	81.5	77.9	83.5	77.0	77.8	82.1	73.9	73.4
**5**	99.8	91.3	98.9	92.8	92.3	94.6	94.1	93.4
**6**	74.2	79.7	72.0	57.6	83.6	76.0	76.6	80.0
**7**	28.8	32.3	47.0	21.5	86.3	91.8	91.4	85.2

Backward locomotor treadmill training); FLTT (forward locomotor treadmill training); N/A-Value not available due to software error. *Subjects BLTT 1 and 2 were poor responders on the 10-meter walk test.

Supplementary Table S3. Interval Activity Level from Training Completion to Follow-Up Timepoints.

	Day 30			Day 90		
**BLTT**	**Types of Activity**	**Hours per week**	**PT in the past week (Y/N)**	**Types of Activity**	**Hours per week**	**PT in the past week (Y/N)**
1	Walking at home, stretching for about 3 hours every day	20	N	Walking, stairs, standing exercise, and arm exercise	10	N
2	Housework, errands, etc. PT, 45 min 1x per week for the last 2 weeks	2	Y	PT and OT, 45min each 3x week. Goes to gym 30 min 3x per week – treadmill, NuStep, arm wheel 10 min each	6	Y
3	PT 1x per week, walking at home	3	Y	1 hr per day exercising at home	7	N
4	"Not much" – walk outside sometimes	1	N	Stretching, walking, swimming in the pool at home	10-15	N
5	Goes to the wellness center 1.5 hr 2x per week	3	N	PT 2x week goes to the aquatic center	4	Y
6	Walks, exercises at home	20	N	Chores at home, walking at home	7	N
7	2 hr per day (5x) walking and exercise at home	10	N	Stretching, resistance bands, weights, walking 30 min per day	15	N
**FLTT**	**Types of Activity**	**Hours per week**	**PT in the past week (Y/N)**	**Types of Activity**	**Hours per week**	**PT in the past week (Y/N)**
1	Goes to PT, iron core (adapted strength and conditioning), exercising at home	5	Y	PT 2x per week, Wheelchair tennis	5	Y
2	Goes golfing ~4 hr per week, ~2 hr mowing lawn/yard work	6	N	Golfing, yard work, household chores	10	N
3	PT 1x per week – strengthening, stairs, walking. Take walks with wife.	5	Y	PT 1x per week, walks outside ~30 min per day on weekdays	4	Y
4	Walking, stationary bike, water therapy 2 hr per week	4	Y	NA	2	N
5	Walking, exercising at home	6	N	Walking, exercising with TheraBand's	6	N
6	1 hr PT per week, walking and arm exercises at home	8	N	1 hr PT 5x per week	5	Y
7	PT and OT 1 hr each, 2x per week. Walking at home and outside	16	Y	PT and OT 1x per week 1.5 hr each. Walking at home.	6	Y

Free form questionnaire regarding interval activity level was administered at the 30- and 90-Day follow-up visits. Backward locomotor treadmill training); FLTT (forward locomotor treadmill training); PT (physical therapy)
